# Mapping QTLs and gene validation studies for Mg^2+^ uptake and translocation using a MAGIC population in rice

**DOI:** 10.3389/fpls.2023.1131064

**Published:** 2023-02-23

**Authors:** Shuai Zhi, Wenli Zou, Jinyan Li, Lijun Meng, Jindong Liu, Jingguang Chen, Guoyou Ye

**Affiliations:** ^1^School of Agriculture, Sun Yat-sen University, Shenzhen, China; ^2^CAAS-IRRI Joint Laboratory for Genomics-Assisted Germplasm Enhancement, Agricultural Genomics Institute in Shenzhen, Chinese Academy of Agricultural Sciences, Shenzhen, China; ^3^Kunpeng Institute of Modern Agriculture at Foshan, Foshan, China; ^4^Rice Breeding Innovations Platform, International Rice Research Institute, Metro Manila, Philippines

**Keywords:** Mg^2+^ uptake, Mg^2+^ translocation, association analysis, quantitative trait loci (QTL), MAGIC population, rice

## Abstract

Magnesium (Mg) is an essential element for plant growth and development. Rice is an important food crop in the world, but there are few studies on the uptake and translocation of Mg^2+^ in rice. We used a multi-parent advanced generation inter-cross (MAGIC) population constructed using four parental lines and genotyped by a 55 K rice SNP array for association analysis to locate QTLs related to Mg^2+^ uptake and translocation in rice at the seedling stage. Four QTLs (*qRMg1, qRMg2, qRMg7* and *qRMg8*) were detected for the root Mg^2+^ concentration, which explained 11.45-13.08% of the phenotypic variation. The Mg^2+^ transporter gene, *OsMGT1*, was within the region of *qRMg1*. Three QTLs (*qSMg3, qSMg7* and *qSMg10*) were detected for the shoot Mg^2+^ concentration, which explained 4.30-5.46% of the phenotypic variation. Two QTLs (*qTrMg3* and *qTrMg8*) were found to affect the translocation of Mg^2+^ from the roots to the shoots, and explained 10.91% and 9.63% of phenotypic variation. *qSMg3* and *qTrMg3* might be the same, since they are very close to each other on chromosome 3. Analysis of candidate genes in the region of *qSMg3* and *qTrMg3* through qRT-PCR, complementation assay in the yeast Mg^2+^ transport-defective mutant CM66, and sequence analysis of the parental lines suggested that *LOC_Os03g04360* may play important roles in Mg^2+^ uptake, translocation and accumulation in rice. Overexpression of *LOC_Os03g04360* can significantly increase the Mg^2+^ concentration in rice seedlings, especially under the condition of low Mg^2+^ supply.

## Introduction

As a key macronutrient, magnesium (Mg) plays an important role in plant growth, development and reproductive success (Verbruggen and Hermans, 2013). The ionic form of magnesium (Mg^2+^) is the most abundant divalent cation in plant cells, and its most prominent role may be its function as a central atom of chloroplast molecules in photosynthesis (Rissler et al., 2002; Cakmak, 2013). Mg is also a necessary element in carbohydrate partitioning (Cakmak et al., 1994; Cakmak, 2013). Lack of magnesium reduces the rate of photosynthesis and disrupts the distribution of carbohydrates from source to sink in plants, while inhibiting the growth of plant organs, eventually leading to a significant decrease in crop quality and productivity (Aitken et al., 1999; Rissler et al., 2002; Verbruggen and Hermans, 2013). The only effective form of Mg absorption by plants is Mg^2+^, which has the smallest ionic radius but the largest hydrated ionic radius in cations (Maguire and Cowan, 2002). This unique chemical property makes Mg^2+^ bind weakly to negative charged soil colloids and root cell walls, which leads to the loss of the exchangeable Mg easily from soil (Aitken et al., 1999; Hermans et al., 2004). Additionally, excessive application of high rates of K^+^ and ${\text{NH}}_{4}^{+}$ fertilizers antagonistically interfere with plant Mg uptake, thus enhances the risk of Mg deficiency (Gransee and Führs, 2013). With the increase of crop yield and multi-cropping, Mg consumption leads to the lack of Mg in soil (Rosanoff, 2013). Therefore, crop production problems caused by magnesium deficiency have gradually become important issues in agriculture.

In view of the unique chemical property and biological significance of Mg^2+^, more and more studies have been conducted to gain understanding of the genetic and molecular mechanism of Mg^2+^ uptake, translocation and distribution in plants (Li et al., 2008; Hermans et al., 2013). MHX (Mg^2+^/H^+^ Exchanger), HKT (High-Affinity K^+^ Transport), CNGC (Cyclic Nucleotide-Gated Channel) and MGT (Magnesium Transporter) have been identified as Mg^2+^ transporters in plants (Shaul et al., 1999; Gebert et al., 2009; Tang and Luan, 2017; Zhang et al., 2017). MHX is a unique vacuolar Mg^2+^ transporter in Arabidopsis (Shaul et al., 1999). OsHKT2;4 has the function of low-affinity Mg^2+^ transporter in rice (Zhang et al., 2017). A CNGC family protein, AtCNGC10, has been indicated to mediate Mg^2+^ influx, particularly in the root meristem and distal elongation zones (Guo et al., 2010). MGT is the best-studied gene family of Mg^2+^ transporter in plants (Gebert et al., 2009; Saito et al., 2013). Although most of the members of the MGT family have Mg transport activity as proven by functional complementation with yeast and bacteria mutants, their physiological roles in plants are largely different. MGT6 can mediate Mg^2+^ uptake in the roots of Arabidopsis (Yan et al., 2018). Two tonoplast-localized transporters, MGT2 and MGT3, are involved in the transport of Mg^2+^ into vacuole in Arabidopsis (Conn et al., 2011). MGT4, MGT5 and MGT9 were found to promote pollen development and male fertility through Mg^2+^ influx in Arabidopsis (Chen et al., 2009; Li et al., 2015; Xu et al., 2015). MGT10 located on the chloroplast envelope membrane regulates Mg^2+^ homeostasis in chloroplasts of Arabidopsis (Drummond et al., 2006; Sun et al., 2017). The expression of *OsMGT1* was highly induced by Mg^2+^ deficiency in shoots, and knockout of *OsMGT1* resulted in a significant reduction in Mg^2+^ content and biomass at seedling stage when grown under Mg-limited conditions (Zhang et al., 2019). Knockout of *OsMGT1* results in decreased Mg^2+^ uptake in the roots by a stable isotope ^25^Mg^2+^ uptake experiment (Chen et al., 2012). This evidence indicates that OsMGT1 is a transporter for root Mg^2+^ uptake in rice. Li et al. (2020) found that a chloroplast-localized Mg^2+^ transporter gene, *OsMGT3*, which is rhythmically expressed in leaf mesophyll cells, partly modulates Mg fluctuations in rice chloroplasts. OsPRR95 and OsPRR59 in rice are transcriptional repressors to negatively regulate the rhythmic expression of *OsMGT3*, which encodes a chloroplast-localized Mg^2+^ transporter (Chen et al., 2022).

As an important food crop in the world, it is of great significance to improve resistance/tolerance to various stresses in rice, including Mg deficiency. Quantitative trait locus (QTL) mapping has been widely used to analyze genetic factors of agronomic traits, including absorption and transport of metal ions (Yang et al., 2018; Gao et al., 2019; Yan et al., 2019). Norton et al. (2010) conducted a multi-element analysis of the leaves and grains of a field-grown rice F_2_ population, and detected eight QTLs for Mg concentration in leaves and five QTLs for Mg concentration in grains. Yang et al. (2018) identified two QTLs for Mg concentration in shoots at the mature stage through a genome-wide association study (GWAS). So far, no major QTLs have been found, fine-mapped or cloned for Mg^2+^ uptake and transport in rice. In this study, we aimed to illuminate the genetic basis of Mg^2+^ uptake and transport at the seedling stage through GWAS with a highly diverse MAGIC population genotyped using a 55 K single nucleotide polymorphisms (SNPs) array.

## Materials and methods

### Plant materials

The MAGIC population used in this experiment was the DC1 population described by Meng et al. (2017). DC1 population was developed by four parental lines A, B, C and D, which came from different countries and had different agronomic characters (**Table S1**). A random sample of 215 lines formed this population were used in this study.

### Plant growth conditions

The paddy rice seeds were surface-sterilized with 10% (v/v) hydrogen peroxide solution for 30 minutes, washed with deionized water and germinated for 48 hours under dark condition and 30°C (Chen et al., 2020). Experiment was laid out according to an augmented randomized complete block design with the four parental lines being replicated in four blocks. A total of 24 seeds per plot were randomly sown in a 96 well PCR plate, with perforations at the bottom of the plate to facilitate the roots to fully contact with the nutrient solution (Naz et al., 2019). The full strength IRRI solution used has the following compositions: 1.0 mM MgSO_4_·7H_2_O, 1.25 mM NH_4_NO_3_, 0.3 mM KH_2_PO_4_, 1.0 mM CaCl_2_, 0.35 mM K_2_SO_4_, 0.5 mM Na_2_SiO_3_, 20.0 μM Fe-EDTA, 20.0 μM H_3_BO_3_, 9.0 μM MnCl_2_, 0.77 μM ZnSO_4_, 0.32 μM CuSO_4_, and 0.39 μM (NH_4_)_6_Mo_7_O_24_, pH 5.5. Rice seedlings were first grown in the 1/4 strength solution for two weeks, and then transferred to the full-strength solution with different Mg^2+^ concentrations for three weeks. The nutrient solution was replaced every three days and the pH was adjusted to 5.5 every day. Plants were grown in a growth room with a 14 h light (30°C) (8:00 – 22:00)/10 h dark (22°C)(22:00 – 8:00) and 60% relative humidity. We explored the Mg^2+^ concentration of four parental lines under normal growth and Mg^2+^ deficiency treatment. After 3-week treatment, the plants were divided into two parts: root and shoot. The tissue samples were dried at 70°C, and 24 seedlings of each line were mixed to measure the dry weight and Mg^2+^ concentration.

### Determination of Mg^2+^ concentration

The dried samples were crushed, and then wet-digested in concentrated HNO_3_ at 120°C for 30 min, and then further digested with HClO_4_ at 180°C until the samples became transparent. Then the samples were diluted with ultrapure water. The Mg^2+^ concentration was determined by inductively coupled plasma mass spectrometry (ICP-MS).

### SNP genotyping and association analysis

Meng et al. (2017) genotyped the MAGIC population with a 55K SNPs array. Selection of high-quality SNPs for QTL mapping used a three-step filtering strategy. First, markers monomorphic among the four parents were removed. Second, set all heterozygous genotypes to “deletion” and delete markers with deletion values greater than 10%. Finally, markers with a minor allele frequency of less than 3% were deleted. The number of markers remaining was 22,160.

The MLM (Mixed Linear Model) implemented in TASSEL version 5.2.3 was used to analyze the associations between SNP markers and traits. *P* < 0.001 was used as the threshold to declare the significance of marker-trait associations. R^2^ was used to evaluate the percentage of phenotypic variance explained of related loci to phenotypic traits.

### RNA extraction and real-time PCR

To examine the expression response of the candidate genes to Mg^2+^ deficiency, seedlings of rice variety Nipponbare or the four parental lines were first grown in the 1/4 strength IRRI solution for two weeks, and then cultured in the full strength IRRI solution with 1.0 mM Mg^2+^ or without Mg^2+^ for three weeks. The roots were sampled for RNA extraction. A randomized complete block design with three replicates and a plot size of 24 seedling were used to layout the experiment. Samples were taken from all the 24 seedlings of a plot and mixed for RNA extraction.

To investigate the expression pattern of the candidate genes in different organs at different growth stages, 3-week-old seedling of Nipponbare precultured hydroponically were transplanted to the paddy field in the Experimenal Farm in Shenzhen of the Agricultural Genomics Institute in Shenzhen, Chinese Academy of Agricultural Sciences. Tissue samples taken includes roots, basal stem, leaf sheath and leaf blade at the vegetative stage and roots, basal stem, lower leaf sheath, lower leaf blade, flag leaf sheath, flag leaf blade, node I–II, inter node II, peduncle, rachis, spikelet, husk and seed at the reproductive stages. A single plant was regarded as a biological replicate and three biological replicates were used.

The total RNA was extracted by Trizol (Vazyme Biotech Co. Ltd, China). Then the total RNA was reverse- transcripted with the HiScript Q RT SuperMix for qPCR kit (Vazyme Biotech Co). The AceQ Universal SYBR qPCR Master Mix kit (Vazyme Biotech Co) was used for quantitative analysis (Chen et al., 2020). The primers for qRT-PCR were shown in **Supplementary Table S2**.

### Expression of candidate genes in yeast

The Mg^2+^ translocation ability of each candidate gene protein was examined by a yeast complementation assay. The yeast mutant CM66, which lacks plasma membrane Mg^2+^ transporters ALR1 and ALR2, was used (Li et al., 2001). The open reading frames of all candidate genes were amplified from the full-length cDNA of rice cv. Nipponbare, and the primer sequences were shown in **Supplementary Table S3**. Each candidate gene was ligated into a pYES2 vector with correct direction.

Empty vector pYES2 and candidate genes vectors were introduced into CM66 yeast cells, respectively, according to the manufacturer’s protocol (Yeast Transformation Kit; Beijing Kulaibo Technology Co. Ltd, China), and transformants were selected on synthetic dextrose medium without uracil (SD-U). Positive clones were cultured in SD-U liquid medium until the early logarithmic phase, concentrated and washed three times with sterile distilled water. After sequential 10-fold dilution, 8 μL of the cell suspension were spotted on SD-U plates containing 1, 4, 64 mmol/L MgCl_2_, respectively. The plates were incubated at 30°C for 3 d before the growth phenotypes were evaluated.

The growth of CM66 yeast strain transformed with various plasmids in liquid SD-U media containing Mg^2+^ was determined. Overnight yeast cells were prepared and the optical density (OD) at 600 nm was adjusted to 0.5 with sterile distilled water. Then, 20 μL of cell suspensions was added to 20 mL liquid SD-U media containing 4, 64, 128 mmol/L MgCl_2_ in each bottle. The OD values at 600 nm were determined at indicated time.

### Sequence analysis of *LOC_Os03g04360, LOC_Os03g04430* and *LOC_Os03g04480* in four parental lines

Seedlings of the four parental lines were used for DNA extraction by the DNeasy Plant Mini Kit (Qiagen, Germany). For each parental line, a single plant was used and the experiment was conducted in triplicate. The DNA samples of the four parental lines were used as templates to amplify the full-length genomic sequence and promoter of *LOC_Os03g04360, LOC_Os03g04430* and *LOC_Os03g04480* by the KOD-FX polymerase (Toyobo, Japan) using specific primers (**Table S4**). After PCR amplification, PCR products were sequenced by the Sangon Biotech Co., Ltd. (Shanghai, China). Sequences of *LOC_Os03g04360, LOC_Os03g04430* and *LOC_Os03g04480* from the four parental lines were aligned and analyzed using MEGA 7.0.

### The development of *LOC_Os03g04360* overexpression transgenic rice

Using the Gateway (Invitrogen) recombi-nation reaction, the open reading frame sequence of *LOC_Os03g04360* was transferred into the destination vector pCAMBIA1300. The vectors were transformed into rice as described previously (Chen et al., 2020).

## Results

### Mg^2+^ concentration of four parental lines and MAGIC population at seedling stage

**Figure 1:**
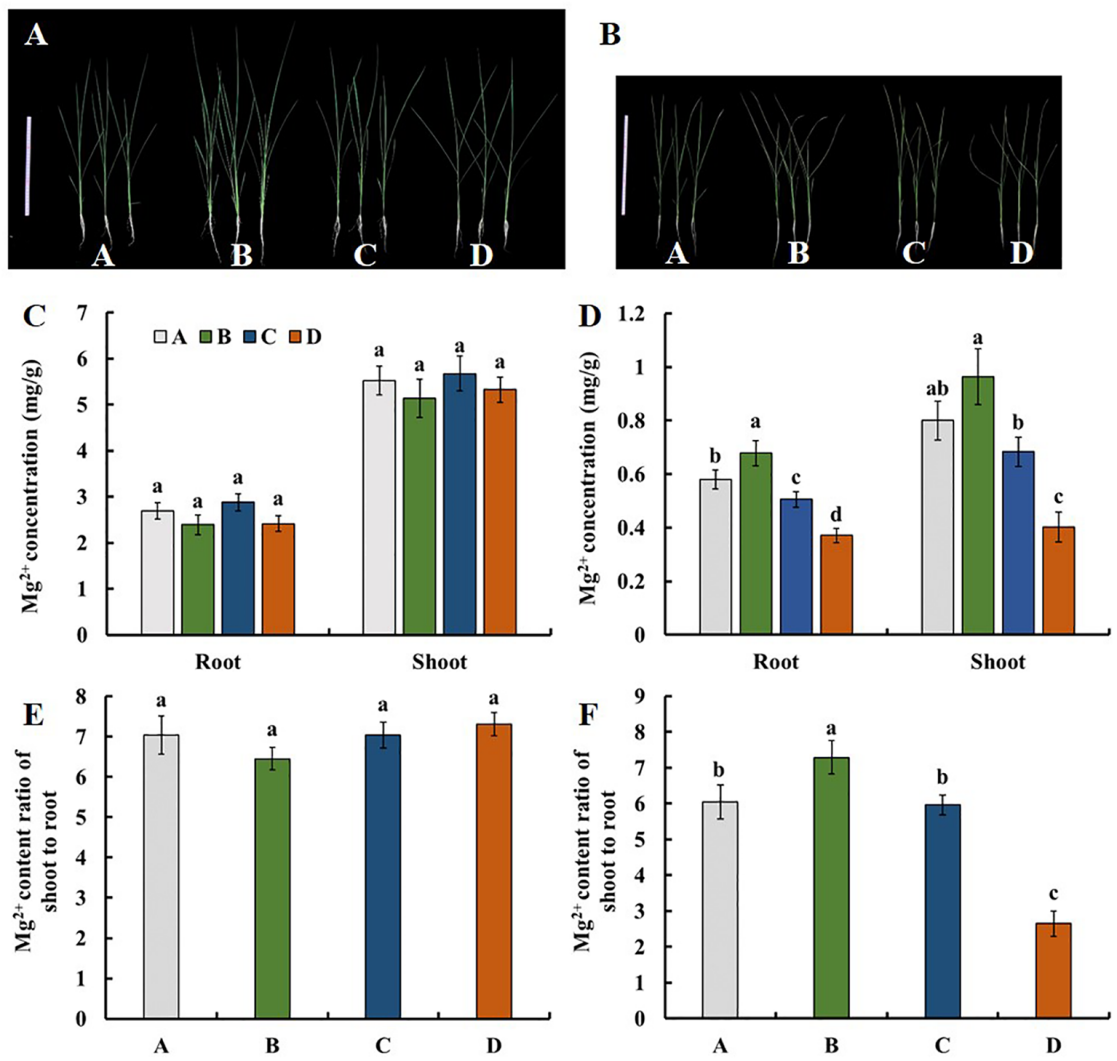
Mg^2+^ uptake and translocation of the four parental lines of the DC1 population. Rice seedlings cultured in the 1/4 strength IRRI solution for two weeks, and in the full strength IRRI solution with **(A)** 1 mM Mg^2+^ or **(B)** without Mg^2+^ for three weeks. Bar: 30 cm. The Mg^2+^ concentration of seedlings treated with **(C)** 1 mM Mg^2+^ or **(D)** without Mg^2+^. The shoot-to-root ratio of the Mg^2+^ content (Mg^2+^ translocation) of seedlings treated with **(E)** 1 mM Mg^2+^ or **(F)** without Mg^2+^. Values are mean ± SE (*n* = 4). The different letters above the bars indicate a significant difference between each line (*P* < 0.01).

**Figure 2:**
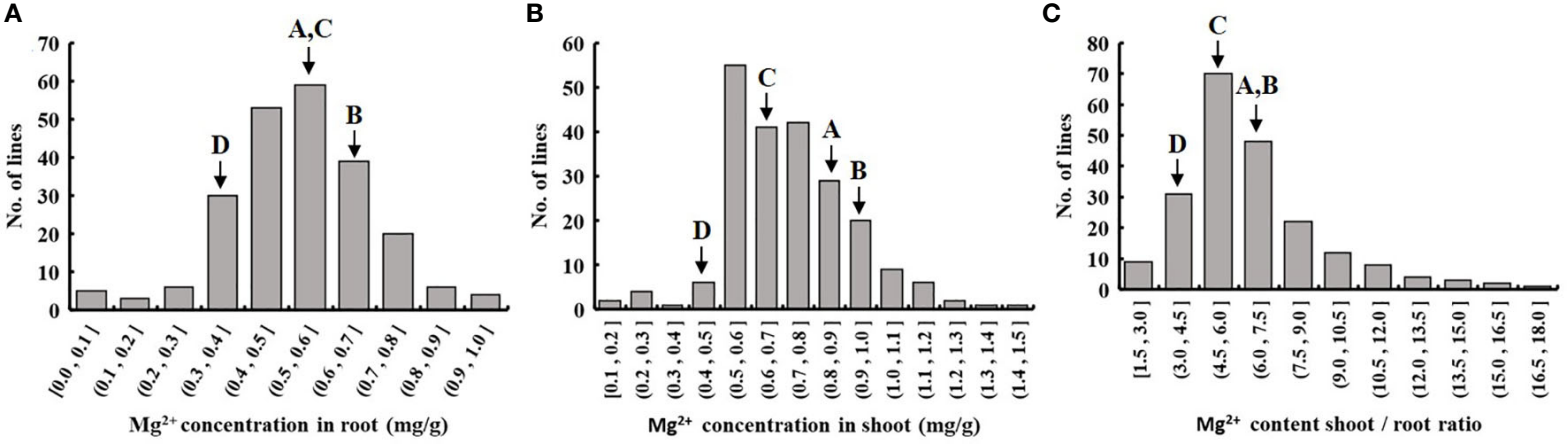
Frequency distribution of Mg^2+^ uptake and translocation traits in the DC1 population. Rice seedlings of the MAGIC population in the 1/4 strength IRRI solution for two weeks, and in the full strength IRRI solution without Mg^2+^ for three weeks. Frequency distribution of **(A)** root Mg^2+^ concentration, **(B)** shoot Mg^2+^ concentration and **(C)** shoot-to-root ratio of Mg^2+^ content. A, B, C and D represent the parental lines A, B, C and D.

We evaluated the Mg^2+^ translocation and accumulation in the four parental lines of the MAGIC DC1 population and its four parental lines. Two-week-old seedlings of the four parental lines with normal growth were treated with 1.0 mM Mg^2+^ (**Figure 1A**) or without Mg^2+^ supply (**Figure 1B**) for three weeks. Under the condition of 1.0 mM Mg^2+^ supply, no significant difference among the four parental lines was found for the Mg^2+^ concentration in roots or shoots (**Figure 1C**), nor for Mg^2+^ translocation (the ratio of Mg^2+^ accumulation in shoots to roots) (**Figure 1E**). After three weeks of Mg^2+^-free treatment, the root and shoot Mg^2+^ concentrations were highest in the parental line B, while the lowest in the parental line D. The root Mg^2+^ concentration of parental line B was 1.83 times that of the parental line D. The shoot Mg^2+^ concentration of B was 2.40 times that of parental line D (**Figure 1D**). The Mg^2+^ translocation of parental line B was the highest, while that of parental line D was the lowest. The Mg^2+^ translocation of parental line B was 2.75 times that of the parental line D (**Figure 1F**). The MAGIC population exhibited significant phenotypic variation for Mg^2+^ concentration in roots and shoots, and for Mg^2+^ translocation (**Table 1**). All the traits displayed normal distributions (**Figure 2**).

**Table 1:** Root and shoot Mg^2+^ concentration of the parental lines and DC1 population.

	A	B	C	D	DC1
Root Mg^2+^ concentration (mg/g)	0.52	0.68	0.55	0.35	0.53 ± 0.15
Shoot Mg^2+^ concentration (mg/g)	0.80	0.94	0.63	0.42	0.67 ± 0.20
Mg^2+^ content shoot/root ratio	6.44	7.03	5.65	3.12	6.42 ± 2.65

### QTLs identified through GWAS

**Figure 3:**
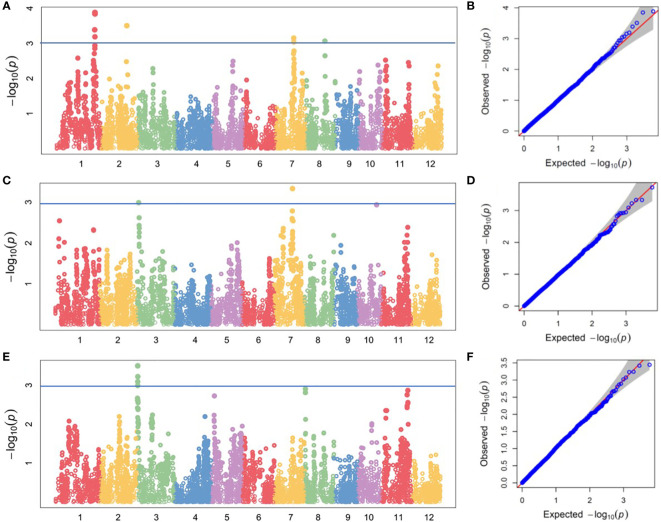
GWAS of magnesium uptake and translocation in the DC1 population. Manhattan plots of for **(A)** root Mg^2+^ concentration, **(C)** shoot Mg^2+^ concentration and **(E)** Mg^2+^ content ratio of shoot to root at seedling stage; Quantile-Quantile plots for **(B)** root Mg^2+^ concentration, **(D)** shoot Mg^2+^ concentration and **(F)** shoot-to-root ratio of Mg^2+^ content.

We performed GWAS analysis for the above traits. Four QTLs for root Mg^2+^ concentration was located on chromosomes 1, 2, 7 and 8, respectively. They explained 13.08%, 12.32%, 11.61% and 11.45% of the phenotypic variation, respectively (**Table 2** and **Figures 3A, B**). Three QTLs were identified for shoot Mg^2+^ concentration, which were distributed on chromosomes 3, 7 and 10, and explained 5.57%, 6.30% and 5.46% of phenotypic variation, respectively (**Table 2** and **Figures 3C, D**). Two QTLs were identified for Mg^2+^ translocation, which were distributed on chromosomes 3 and 8, and explained 10.91% and 9.63% of phenotypic variation, respectively (**Table 2** and **Figures 3E, F**).

**Table 2:** QTLs for Mg^2+^ uptake and translocation detected in the DC1 MAGIC population.

QTLs	Alleles	Chr.	Position (bp)	P-value	R^2^(%)	Gene Symbol	References
Root Mg^2+^ concentration							
*qRMg1*	C/T	1	37477188	1.32×10^-4^	13.08	*OsMGT1*	Chen et al., 2012; Zhang et al., 2019
*qRMg2*	C/T	2	24697144	4.08×10^-4^	12.32		
*qRMg7*	G/A	7	17667136	7.02×10^-4^	11.61		
*qRMg8*	G/A	8	18377025	8.55×10^-4^	11.45		
Shoot Mg^2+^ concentration							
*qSMg3*	A/G	3	1933557	1.03×10^-3^	5.57		
*qSMg7*	G/T	7	17840418	4.64×10^-4^	6.30		
*qSMg10*	T/C	10	18784637	1.16×10^-3^	5.46		
Mg^2+^ content ratio of shoot to root							
*qTrMg3*	C/T	3	2110365	3.17×10^-4^	10.91		
*qTrMg8*	A/G	8	723255	1.24×10^-3^	9.63		

SNP position is based on rice genome reference sequence MSU V 7.0. Peaks exhibiting significance threshold level within a physical distance of 1.0Mb were delineated into a single QTL. Chr.: Chromosome; R^2^ (%): Phenotypic variance explained.

### Identification of the candidate genes on chromosome 3

**Figure 4:**
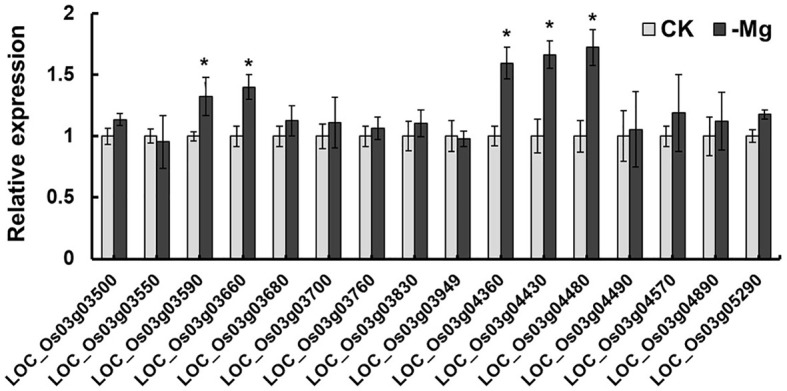
Relative expression of candidate genes for *qSMg3 and qTrMg3* under different Mg^2+^ concentrations. The rice cv. Nipponbare seedlings in the 1/4 strength IRRI solution for two weeks, and in IRRI solution with 1 mM Mg^2+^ (CK) or without Mg^2+^ (-Mg) for three weeks. RNA was extracted from rice roots. Values are mean ± SE (n = 3). * A significant difference at the 0.01 level.

The chromosome 3 has a region harboring QTLs for two traits. They are *qSMg3* for shoot Mg^2+^ concentration and *qTrMg3* for Mg^2+^ translocation. Through annotation (http://rice.plantbiology.msu.edu/index.shtml) and literature information, 16 genes were chosen as the candidate genes responsible for Mg^2+^ translocation and accumulation. Among them, eight encode proteins with transmembrane structure (**Figure S1**), including *OsMGT9 (LOC_Os03g04480)*, a member of MGT family (**Table 3**). Five genes including *LOC_Os03g03590, LOC_Os03g03660, LOC_Os03g04360, LOC_Os03g04430* and *LOC_Os03g04480* were found to be induced by Mg^2+^ deficiency (**Figure 4**).

**Table 3:** Annotations of the selected candidate genes for *qSMg3* and *qTrMg3*.

Gene	MSU ID	Annotation
1	LOC_Os03g03500	heavy metal-associated domain containing protein, expressed
2	LOC_Os03g03550	bZIP family transcription factor, putative, expressed
3	LOC_Os03g03590	transporter, monovalent cation:proton antiporter-2 family, putative, expressed
4	LOC_Os03g03660	CAMK_like.17 - CAMK includes calcium/calmodulin depedent protein kinases, expressed
5	LOC_Os03g03680	transporter family protein, putative, expressed
6	LOC_Os03g03700	MLO domain containing protein, putative, expressed
7	LOC_Os03g03760	MYB family transcription factor, putative, expressed
8	LOC_Os03g03830	EF hand family protein, putative, expressed
9	LOC_Os03g03949	lectron carrier/protein disulfide oxidoreductase, putative, expressed
10	LOC_Os03g04360	inorganic phosphate transporter, putative, expressed
11	LOC_Os03g04430	protein phosphatase 2C, putative, expressed
12	LOC_Os03g04480	CorA-like magnesium transporter protein, putative, expressed
13	LOC_Os03g04490	cyclin-dependent kinase inhibitor, putative, expressed
14	LOC_Os03g04570	peptide transporter PTR3-A, putative, expressed
15	LOC_Os03g04890	zinc finger family protein, putative, expressed
16	LOC_Os03g05290	aquaporin protein, putative, expressed

**Figure 5:**
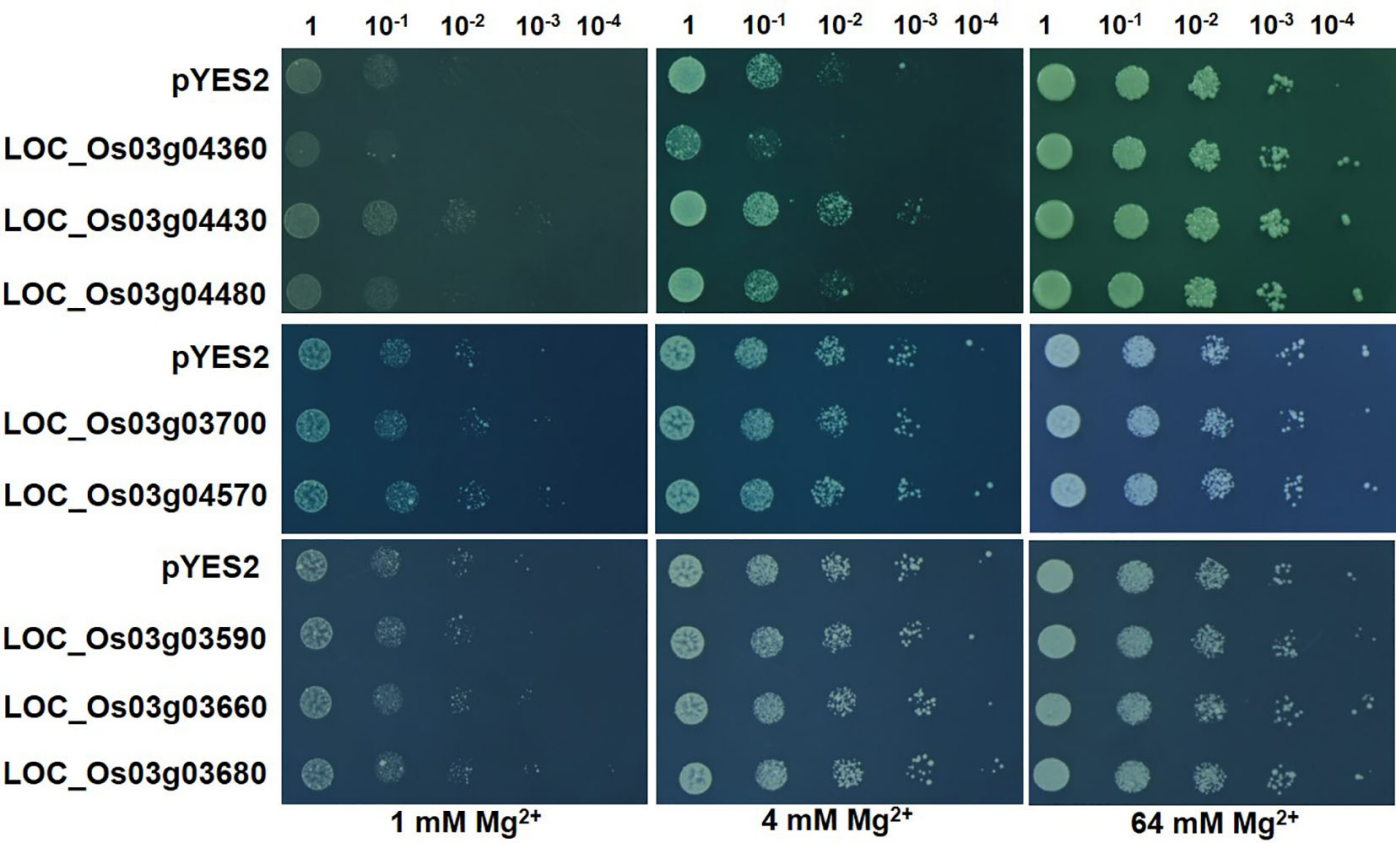
Expression of the candidate genes in yeast mutant CM66. Overnight yeast cell suspension of CM66 transformed with empty vector *pYES2* or candidate genes were serially diluted (1:10) and spotted on the solid media containing 1, 4 or 64 mmol/L MgSO_4_. Pictures were taken after four days growth at 30°C.

The five genes responsive to Mg^2+^ deficiency and three other genes with complete transmembrane structure (*LOC_Os03g03680, LOC_Os03g03700, LOC_Os03g04570*) were expressed in the yeast mutant CM66. Under the condition of 1 or 4 mmol/L Mg^2+^ supply under solid medium for four days, *LOC_Os03g04430* increased the growth of CM66 and while *LOC_Os03g04360* inhibited (**Figure 5**). The other seven genes, including *OsMGT9* (*LOC_Os03g04480*), did not show any effects on the growth of CM66 (**Figure 5**). The experiment of liquid medium with different Mg^2+^ concentrations confirmed that the growth of CM66 under 4 mmol/L Mg^2+^ supply was increased by *LOC_Os03g04430*, inhibited by *LOC_Os03g04360* (**Figure S2**).

### The variation of promoter sequence leads to the expression difference of *LOC_Os03g04360* in four parental lines

**Figure 6:**
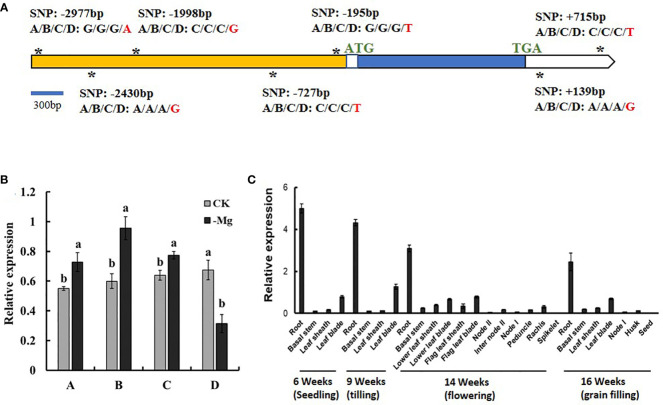
Sequence and expression of *LOC_Os03g04360* in the four parental lines of the DC1 population. **(A)** Gene structure of *LOC_Os03g04360* and polymorphism locations (SNPs, asterisks). The SNPs are underlined with asterisks. **(B)** Relative expression of *LOC_Os03g04360* under different Mg^2+^ concentrations. The rice seedlings grown in the 1/4 strength IRRI solution for two weeks, and in the full strength IRRI solution with 1 mM Mg^2+^ (CK) or without Mg^2+^ (-Mg) for three weeks. RNA was extracted from rice roots. **(C)** Expression of *LOC_Os03g04360* in different organs at different growth stages. RNA Samples were taken from cv. Nipponbare grown in a paddy field. Values are mean ± SE (n = 3). The different letters above the bars indicate significant difference between the control and treatments at *P* < 0.01.

To further screen candidate genes to obtain the target genes, the sequences of *LOC_Os03g04360*, *LOC_Os03g04430* and *LOC_Os03g04480* of the four parental lines were analyzed by PCR amplification and sequencing. The coding region sequence of *LOC_Os03g04360*, *LOC_Os03g04430* and *LOC_Os03g04480*, including exon and intron, had no difference among the four parental lines (**Figures 6A**, **S4A, B**). There was no difference among A, B and C in the 3000 bp region of the upstream of the ATG and the 3’-UTR sequence after the TGA for both of *LOC_Os03g04360* and *LOC_Os03g04430* (**Figures 6A**, **S3A**). Compared with A, B and C, D has five single-nucleotide mutations in the 3000 bp region of the upstream of the ATG and two single-nucleotide mutations in the 3’-UTR of *LOC_Os03g04360* (**Figure 6A**), plus a single-nucleotide mutation in the 3000 bp upstream of the ATG of *LOC_Os03g04430* (**Figure S3A**). At the same time, we analyzed the gene sequence of *LOC_Os03g04480* of the four parental lines, and found no natural variation (**Figure S3B**).

The expression patterns of *LOC_Os03g04360*, *LOC_Os03g04430* and *LOC_Os03g04480* in the four parental lines are shown in **Figures 6B**, **S3C, D**. Compared with the normal Mg^2+^ supply, the expression of *LOC_Os03g04360* was significantly induced by Mg^2+^ deficiency in A, B and C while significantly reduced in D (**Figure 6B**). Compared with the normal Mg^2+^ supply, the expression level of *LOC_Os03g04430* was increased under Mg^2+^ deficiency in all the four parental lines (**Figure S3C**). *LOC_Os03g04480* was significantly induced by Mg^2+^ deficiency in all parental lines (**Figure S3D**). Therefore, *LOC_Os03g04430* is not considered as a candidate gene underlying *qSMg3* and *qTrMg3*.

In order to further analyze the biological functions of *LOC_Os03g04360*, its expression in different organs at different growth stages were investigated. *LOC_Os03g04360* strongly expressed in root at different growth stages, but only weakly expressed in other tissues (**Figure 6C**).

### Mg^2+^ accumulation of *LOC_Os03g04360* overexpression transgenic plants under different Mg^2+^ supply at seedling stage

**Figure 7:**
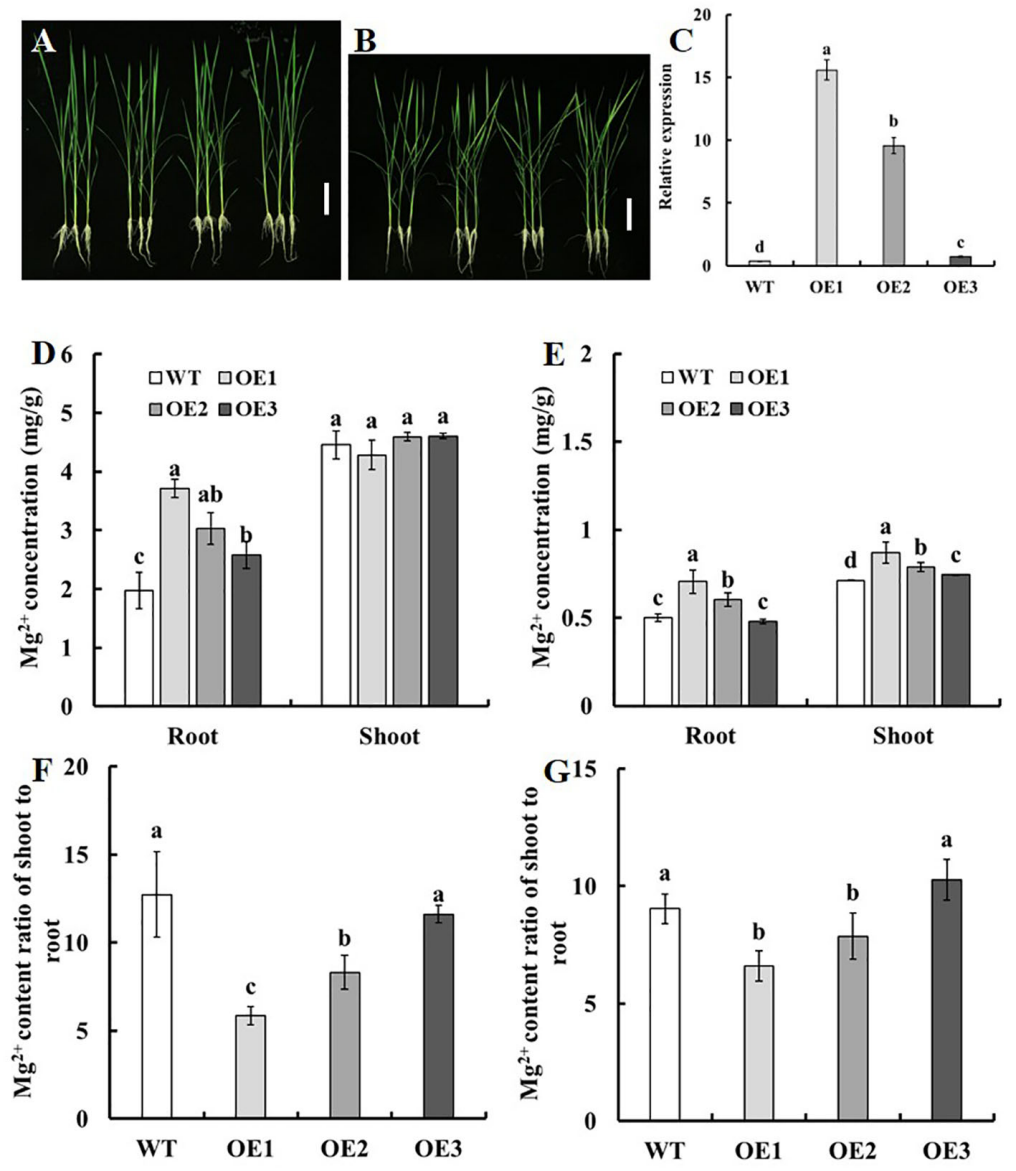
Mg^2+^ uptake and translocation of the *LOC_Os03g04360* overexpression lines. Two-week-old seedlings of the transgenic lines and WT with normal growth were treated with 250 μM Mg^2+^ or 10 μM Mg^2+^ supply for two weeks. **(A, B)** Phenotype of the *LOC_Os03g04360* overexpression lines grown with **(A)** 250 μM Mg^2+^ and **(B)** 10 μM Mg^2+^ supply. **(C)** Quantitative real-time PCR analysis of endogenous *LOC_Os03g04360* expression in various transgenic lines and WT. RNA was collected from root. The Mg^2+^ concentration in roots and shoots in *LOC_Os03g04360* overexpression lines and WT under **(D)** 250 μM Mg^2+^ or **(E)** 10 μM Mg^2+^ supply. The shoot-to-root ratio of the Mg^2+^ content of seedlings treated with **(F)** 250 μM Mg^2+^ or **(G)** 10 μM Mg^2+^. Error bars: SE (n = 3 plants). The different letters above the bars indicate a significant difference between each line (*P* < 0.01).

Analysis of candidate genes in the region of *qSMg3* and *qTrMg3* through qRT-PCR, complementation assay in the yeast Mg^2+^ transport-defective mutant CM66, and sequence analysis of the parental lines suggested that *LOC_Os03g04360* may play important roles in Mg^2+^ uptake, translocation and accumulation in rice. We introduced *pUbi : LOC_Os03g04360* expression construct into cv. Nipponbare (WT) using agrobacterium tumefaciens-mediated transformation. The expression of *LOC_Os03g04360* in roots increased 26-42-fold in OE1 and OE2 compared with WT, but only 1-fold in OE3 (**Figure 7C**).

We further analyzed the effect of overexpression of *LOC_Os03g04360* on Mg^2+^ accumulation at seedling stage. Two-week-old seedlings of the transgenic lines and WT with normal growth were treated with 250 μM Mg^2+^ (**Figure 7A**) or 10 μM Mg^2+^ supply (**Figure 7B**) for two weeks. Under the condition of 250 μM Mg^2+^ supply, no significant difference among the transgenic lines and WT was found for the Mg^2+^ concentration in shoots (**Figure 7D**), compared with WT, the Mg^2+^ concentration in roots of OE1, OE2 and OE3 increased by 87.7%, 53.4% and 30.5% respectively (**Figure 7D**). Under the condition of 10 μM Mg^2+^ supply, compared with WT, the Mg^2+^ concentration in roots of OE1, OE2 and OE3 increased by 40.6%, 20.2% and 0% respectively, and that in shoots increased by 21.9%, 10.5% and 5.4% respectively (**Figure 7E**). Under the two treatments, the Mg^2+^ content ratio of shoot to root of OE1 and OE2 were significantly reduced, and there was no difference between OE3 and WT (**Figures 7F, G**).

## Discussion

### QTLs for Mg^2+^ uptake and translocation

High-resolution mapping of rice by using MAGIC populations has been previously reported (Bandillo et al., 2013; Meng et al., 2016a; Meng et al., 2016b; Meng et al., 2017). We previously identified QTLs associated with the toxicity tolerance of rice to three essential metals (Fe, Zn, and Al) by using three MAGIC populations including DC1, DC2 and 8way populations (Meng et al., 2017). The four parents of the MAGIC DC1 population used in this study displayed substantial differences in Mg^2+^ uptake, translocation and accumulation under Mg^2+^ deficiency (**Figure 1** and **Table 1**). In this study, we aimed to identify the loci related to Mg^2+^ uptake and transport in the seedling stage by screening the MAGIC DC1 population in a hydroponic system, using a 55K SNPs array, and using a mixed linear model to conduct association analysis.

In the present study, four QTLs related to the concentration of Mg^2+^ in roots, three QTLs related to the shoot Mg^2+^ concentration, and two QTLs related to the Mg^2+^ translocation were identified (**Table 2**). These QTLs did not co-locate with the QTLs of Mg concentration in leaves or shoots at the mature stage in the field of Norton et al. (2010) and Yang et al. (2018), which may be caused by differences in growth conditions and growth stages. The *qRMg1* (Chr.1: 37.5 Mb) was located 187 kb away from the *OsMGT1*. *OsMGT1* is a plasma membrane-localized transporter, and has high expression in root tips and vascular tissues (Chen et al., 2012), overexpression of *OsMGT1* increased Mg^2+^ content under low-Mg^2+^ supply (Zhang et al., 2019).

### *LOC_Os03g04360* could be a novel functional gene controlling Mg^2+^ uptake and translocation in rice

A set of candidate genes for *qSMg3* and *qTrMg3* located on the same region of chromosome 3 were studied in the present study (**Table 2**).

*LOC_Os03g04360*, was found to be responsive to Mg^2+^ deficiency (**Figure 4**) and affected the growth of CM66 under low Mg^2+^ condition (**Figures 5**, **S3**). *LOC_Os03g04360* inhibited the growth of CM66 under low Mg^2+^ supply (**Figures 5**, **S3**). *LOC_Os03g04360* belongs to the phosphate transporter gene family *OsPHT1* (Chiou and Lin, 2011; Secco et al., 2013). The PHT1 family promotes both Pi uptake in soil and Pi transfer in plants (Ai et al., 2009). PHT1 family members also participate in other biological processes. *PvPht1;4*, *OsPT4*, OsPT8 and *PHT1;1* participate in the uptake and accumulation of As (V) by plants (Catarecha et al., 2007; Wang et al., 2016; Cao et al., 2017; Ye et al., 2017; Sun et al., 2020). *OsPT8* also regulates disease resistance by regulating rice mitogen-resistant protein interaction factor kinase BWMK1 (Dong et al., 2019). Phosphate transporter OsPHT1;8 was involved in auxin signal transduction in rice roots (Jia et al., 2017). The coding region sequence of *LOC_Os03g04360* had no difference among the four parental lines (**Figure 6A**). Compared with A, B and C, the parental line D showed five single-nucleotide mutations in the 3000 bp region of the upstream of ATG, and two single-nucleotide mutations in the 3’-UTR of *LOC_Os03g04360* (**Figure 6A**). It is well-known that variation of promoter sequence of a gene can change its expression and function. The natural variation of the *GSE5* promoter contributes to the grain size diversity of rice (Duan et al., 2017). Natural variation in *OsCBL10* promoter can affect flood tolerance during seed germination of rice subspecies (Ye et al., 2018). Natural variation in *OsHMA3* promoter contributes to differential grain cadmium accumulation between *Indica* and *Japonica* rice (Liu et al., 2020). Our results that Mg^2+^ deficiency significantly induced the expression of *LOC_Os03g04360* in A, B and C but inhibited it in D (**Figure 6B**) could be caused by the observed variations in the promoter. The protein of *LOC_Os03g04360* may have Mg^2+^ transport activity and participate in the Mg^2+^ uptake and translocation process in rice.

We introduced *pUbi : LOC_Os03g04360* expression construct into Nipponbare using agrobacterium tumefaciens-mediated transformation. It was found that overexpression of *LOC_Os03g04360* could significantly increase the Mg^2+^ concentration in rice roots under different Mg^2+^ supply at seedling stage (**Figures 7D, E**), but decreased Mg^2+^ content ratio of shoot to root (**Figures 7F, G**). Dai et al. (2022) showed that LOC_Os03g04360 was located in the plasma membrane of cells. *LOC_Os03g04360* was strongly expressed in roots at different growth stages, but weakly expressed in other tissues (**Figure 6C**). Taken together, the present results suggested that *LOC_Os03g04360* may play an important role in the uptake of Mg^2+^ in roots and the translocation of Mg^2+^ from root to shoot. It means that *LOC_Os03g04360* may promote the uptake and accumulation of Mg^2+^ and inhibit translocation in rice. Further characterizations of *LOC_Os03g04360* is needed to elucidate its functional significance in Mg^2+^ uptake, translocation and accumulation in rice.

## Data availability statement

The original contributions presented in the study are included in the article/**Supplementary Material**. Further inquiries can be directed to the corresponding authors.

## Author contributions

Conceived and designed the experiments: JC and GY. Performed the experiments: SZ, JC, WZ, JinyL, LM and JindL. Analyzed the data: SZ, JC and JindL. Wrote and revised the paper: JC, GY and SZ. All authors contributed to the article and approved the submitted version.

## Supplementary material

Supplementary Table 1 Description of the four parental lines used for developing the DC1 population. GID: germplasm identification number used in the International Rice Information System (http://irri.org/).

Supplementary Figure 1 Topological model of proteins encoded by candidate genes. The probability of transmembrane location predicted by the TMHMM algorithm (https://services.healthtech.dtu.dk/service.php?TMHMM-2.0). Red areas indicate predicted TM spans.

Supplementary Figure 2 Expression of the candidate genes in yeast mutant CM66 in liquid medium. Yeast strains were grown in liquid media with **(A)** 4mmol/L MgSO_4_, **(B)** 64 mmol/L MgSO4, or **(B)** 128 mmol/L MgSO_4_ for 75 h. The absorbance at 600 nm (OD600) of cell cultures was measured every 5 h. Values are mean ± SE (*n* = 3). The asterisks above the bars indicate significant difference between lines at *P* < 0.01.

Supplementary Figure 3 Sequence and expression of *LOC_Os03g04430* and *LOC_Os03g04480* in the four parental lines of the DC1 population. Gene structure of **(A)** *LOC_Os03g04430* and **(B)** *LOC_Os03g04480* and polymorphism locations (SNPs, asterisks). The SNPs are underlined with asterisks. Relative expression of **(C)** *LOC_Os03g04430* and **(D)** *LOC_Os03g04480* under different Mg^2+^ concentrations. The rice seedlings in the 1/4 strength IRRI solution for two weeks, and in the full strength IRRI solution with 1 mM Mg^2+^ (CK) or without Mg^2+^ (-Mg) for three weeks. RNA was extracted from rice roots. Values are mean ± SE (n = 3). The different letters above the bars indicate significant difference between the control and treatments at *P* < 0.01.
