# Supplementary material for: Mapping QTLs and gene validation studies for Mg2+ uptake and translocation using a MAGIC population in rice
Source: Front Plant Sci. 2023 Feb 23;14:1131064. doi: 10.3389/fpls.2023.1131064 (PMC9996051; doi:10.3389/fpls.2023.1131064)
Supplement: Supplementary Table 1 — Description of the four parental lines used for developing the DC1 population. GID: germplasm identification number used in the International Rice Information System (http://irri.org/). [file Presentation_1.pptx]

## Slide 1
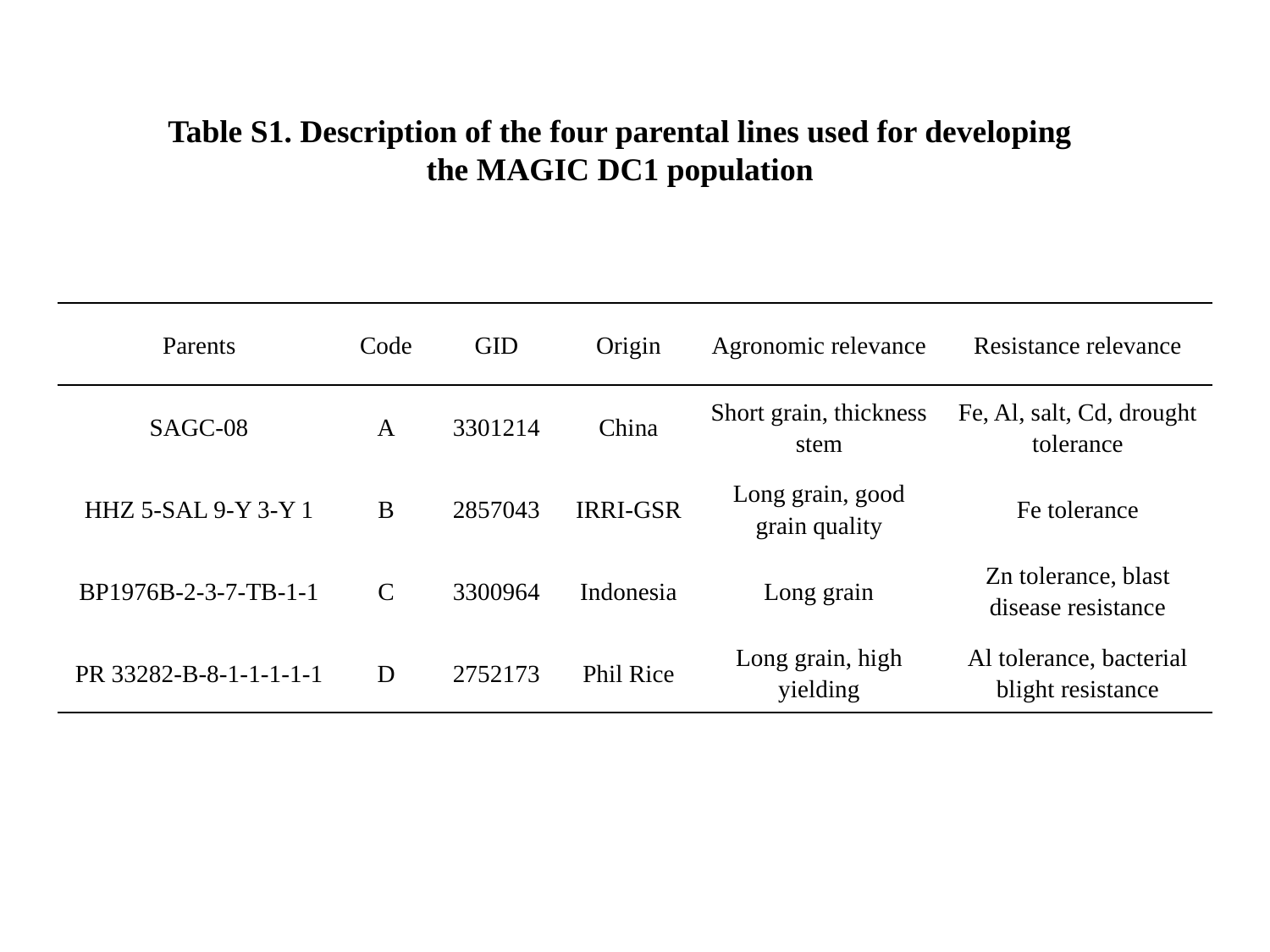

Table S1. Description of the four parental lines used for developing the MAGIC DC1 population
| Parents | Code | GID | Origin | Agronomic relevance | Resistance relevance |
| --- | --- | --- | --- | --- | --- |
| SAGC-08 | A | 3301214 | China | Short grain, thickness stem | Fe, Al, salt, Cd, drought tolerance |
| HHZ 5-SAL 9-Y 3-Y 1 | B | 2857043 | IRRI-GSR | Long grain, good grain quality | Fe tolerance |
| BP1976B-2-3-7-TB-1-1 | C | 3300964 | Indonesia | Long grain | Zn tolerance, blast disease resistance |
| PR 33282-B-8-1-1-1-1-1 | D | 2752173 | Phil Rice | Long grain, high yielding | Al tolerance, bacterial blight resistance |

## Slide 2
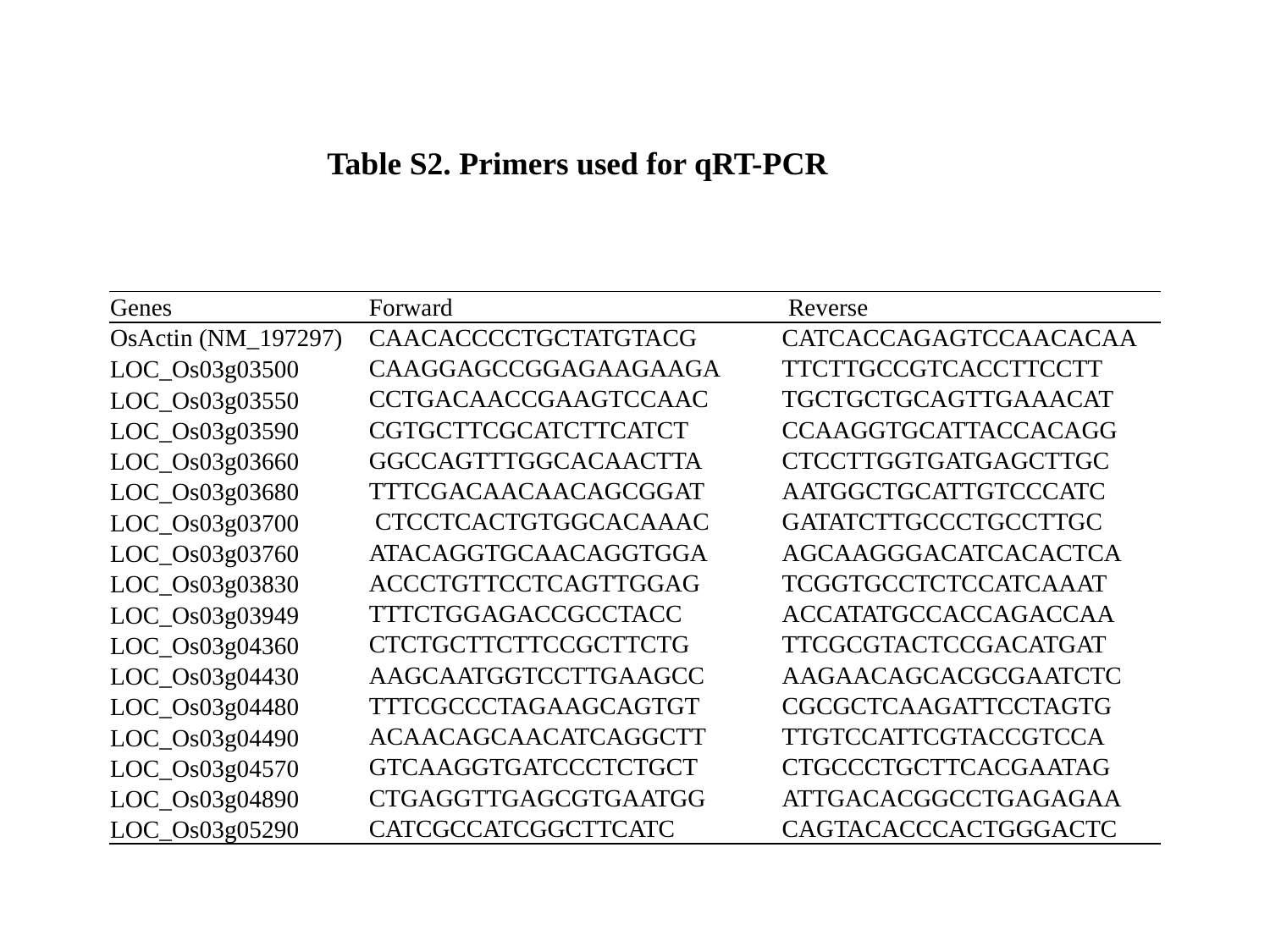

Table S2. Primers used for qRT-PCR
| Genes | Forward | Reverse |
| --- | --- | --- |
| OsActin (NM\_197297) | CAACACCCCTGCTATGTACG | CATCACCAGAGTCCAACACAA |
| LOC\_Os03g03500 | CAAGGAGCCGGAGAAGAAGA | TTCTTGCCGTCACCTTCCTT |
| LOC\_Os03g03550 | CCTGACAACCGAAGTCCAAC | TGCTGCTGCAGTTGAAACAT |
| LOC\_Os03g03590 | CGTGCTTCGCATCTTCATCT | CCAAGGTGCATTACCACAGG |
| LOC\_Os03g03660 | GGCCAGTTTGGCACAACTTA | CTCCTTGGTGATGAGCTTGC |
| LOC\_Os03g03680 | TTTCGACAACAACAGCGGAT | AATGGCTGCATTGTCCCATC |
| LOC\_Os03g03700 | CTCCTCACTGTGGCACAAAC | GATATCTTGCCCTGCCTTGC |
| LOC\_Os03g03760 | ATACAGGTGCAACAGGTGGA | AGCAAGGGACATCACACTCA |
| LOC\_Os03g03830 | ACCCTGTTCCTCAGTTGGAG | TCGGTGCCTCTCCATCAAAT |
| LOC\_Os03g03949 | TTTCTGGAGACCGCCTACC | ACCATATGCCACCAGACCAA |
| LOC\_Os03g04360 | CTCTGCTTCTTCCGCTTCTG | TTCGCGTACTCCGACATGAT |
| LOC\_Os03g04430 | AAGCAATGGTCCTTGAAGCC | AAGAACAGCACGCGAATCTC |
| LOC\_Os03g04480 | TTTCGCCCTAGAAGCAGTGT | CGCGCTCAAGATTCCTAGTG |
| LOC\_Os03g04490 | ACAACAGCAACATCAGGCTT | TTGTCCATTCGTACCGTCCA |
| LOC\_Os03g04570 | GTCAAGGTGATCCCTCTGCT | CTGCCCTGCTTCACGAATAG |
| LOC\_Os03g04890 | CTGAGGTTGAGCGTGAATGG | ATTGACACGGCCTGAGAGAA |
| LOC\_Os03g05290 | CATCGCCATCGGCTTCATC | CAGTACACCCACTGGGACTC |

## Slide 3
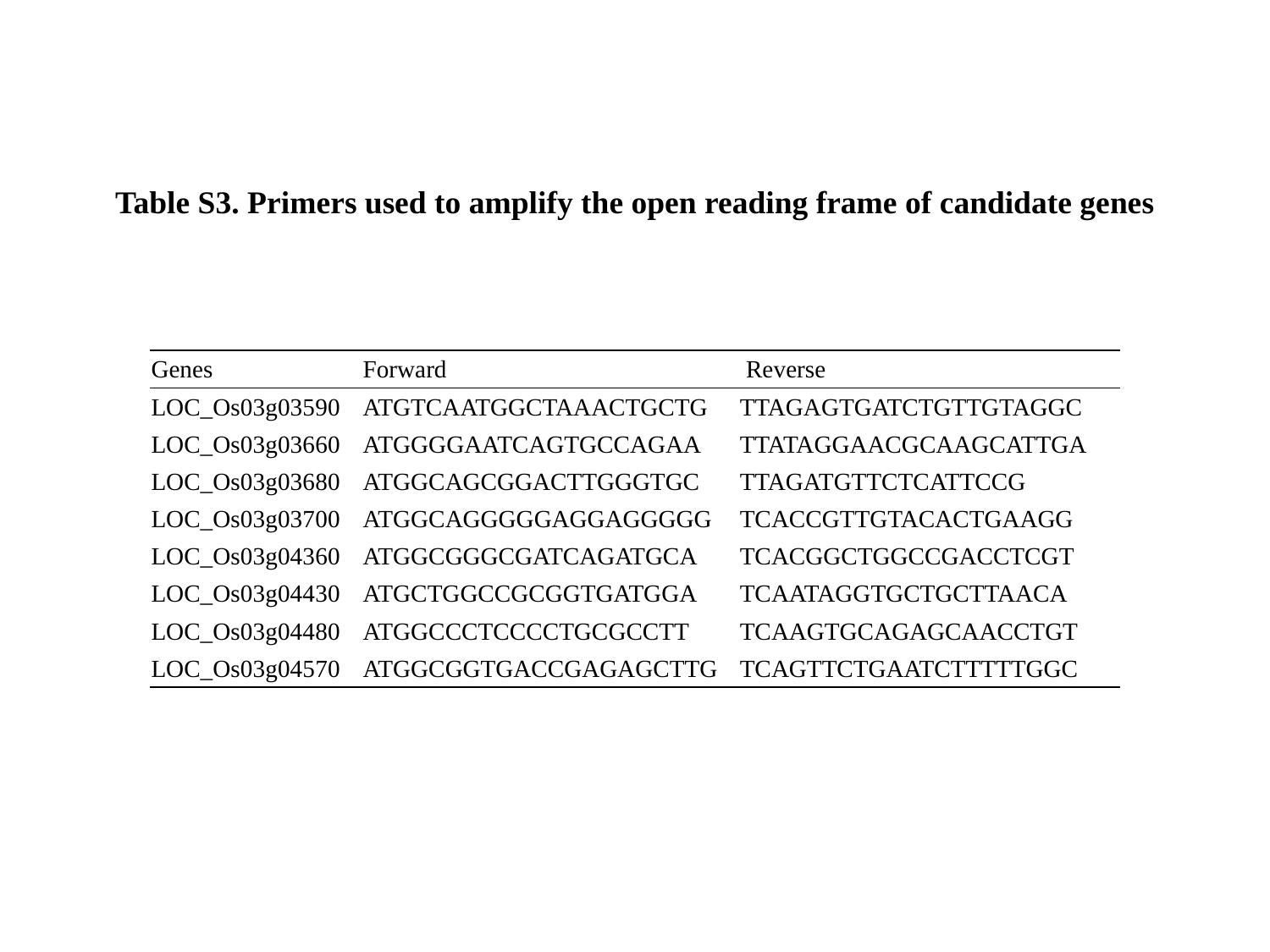

Table S3. Primers used to amplify the open reading frame of candidate genes
| Genes | Forward | Reverse |
| --- | --- | --- |
| LOC\_Os03g03590 | ATGTCAATGGCTAAACTGCTG | TTAGAGTGATCTGTTGTAGGC |
| LOC\_Os03g03660 | ATGGGGAATCAGTGCCAGAA | TTATAGGAACGCAAGCATTGA |
| LOC\_Os03g03680 | ATGGCAGCGGACTTGGGTGC | TTAGATGTTCTCATTCCG |
| LOC\_Os03g03700 | ATGGCAGGGGGAGGAGGGGG | TCACCGTTGTACACTGAAGG |
| LOC\_Os03g04360 | ATGGCGGGCGATCAGATGCA | TCACGGCTGGCCGACCTCGT |
| LOC\_Os03g04430 | ATGCTGGCCGCGGTGATGGA | TCAATAGGTGCTGCTTAACA |
| LOC\_Os03g04480 | ATGGCCCTCCCCTGCGCCTT | TCAAGTGCAGAGCAACCTGT |
| LOC\_Os03g04570 | ATGGCGGTGACCGAGAGCTTG | TCAGTTCTGAATCTTTTTGGC |

## Slide 4
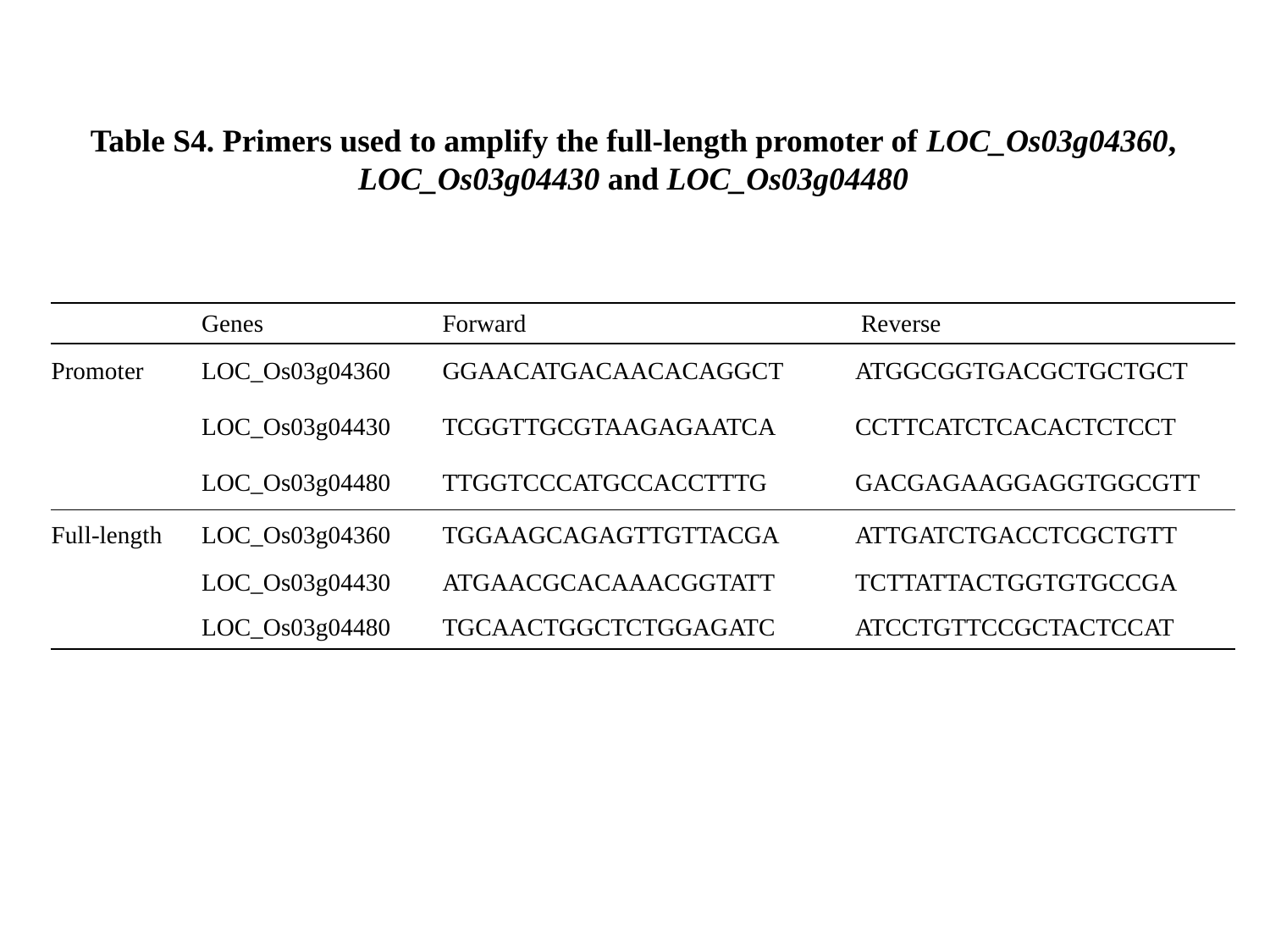

Table S4. Primers used to amplify the full-length promoter of LOC_Os03g04360, LOC_Os03g04430 and LOC_Os03g04480
| | Genes | Forward | Reverse |
| --- | --- | --- | --- |
| Promoter | LOC\_Os03g04360 | GGAACATGACAACACAGGCT | ATGGCGGTGACGCTGCTGCT |
| | LOC\_Os03g04430 | TCGGTTGCGTAAGAGAATCA | CCTTCATCTCACACTCTCCT |
| | LOC\_Os03g04480 | TTGGTCCCATGCCACCTTTG | GACGAGAAGGAGGTGGCGTT |
| Full-length | LOC\_Os03g04360 | TGGAAGCAGAGTTGTTACGA | ATTGATCTGACCTCGCTGTT |
| | LOC\_Os03g04430 | ATGAACGCACAAACGGTATT | TCTTATTACTGGTGTGCCGA |
| | LOC\_Os03g04480 | TGCAACTGGCTCTGGAGATC | ATCCTGTTCCGCTACTCCAT |

## Slide 5
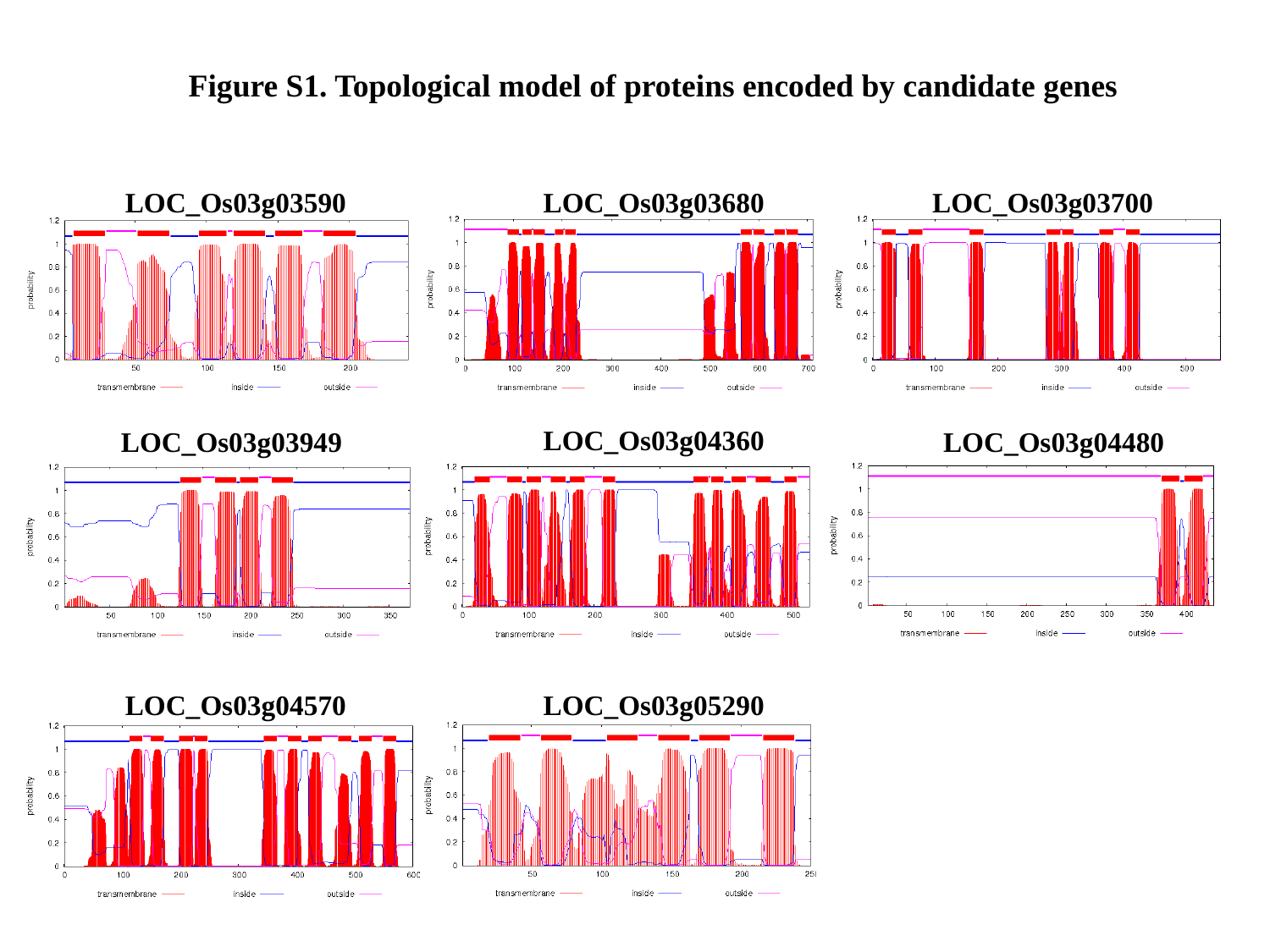

Figure S1. Topological model of proteins encoded by candidate genes
LOC_Os03g03590
LOC_Os03g03700
LOC_Os03g03680
LOC_Os03g04360
LOC_Os03g03949
LOC_Os03g04480
LOC_Os03g04570
LOC_Os03g05290

## Slide 6
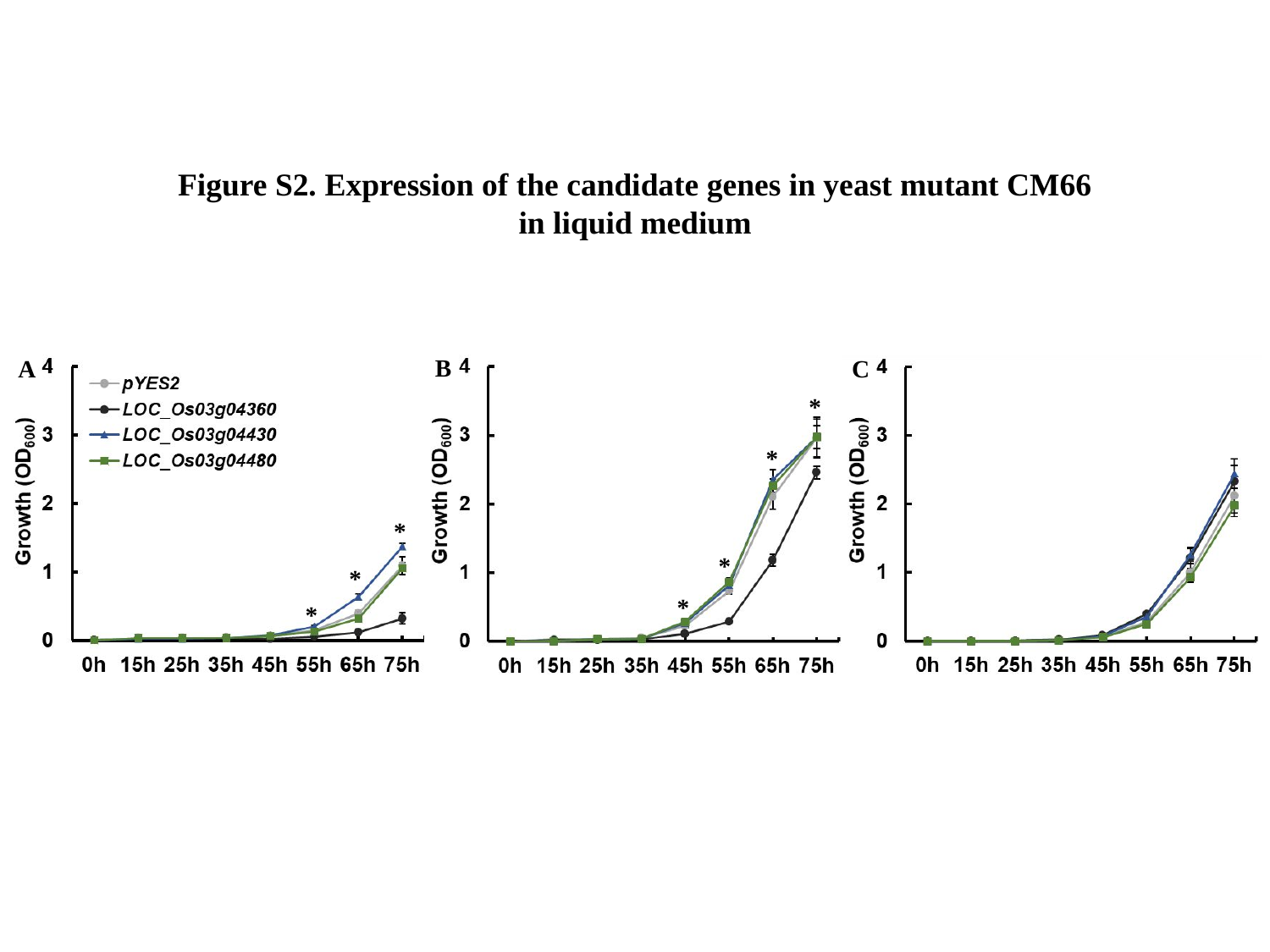

Figure S2. Expression of the candidate genes in yeast mutant CM66 in liquid medium
B
A
C
*
*
*
*
*
*
*

## Slide 7
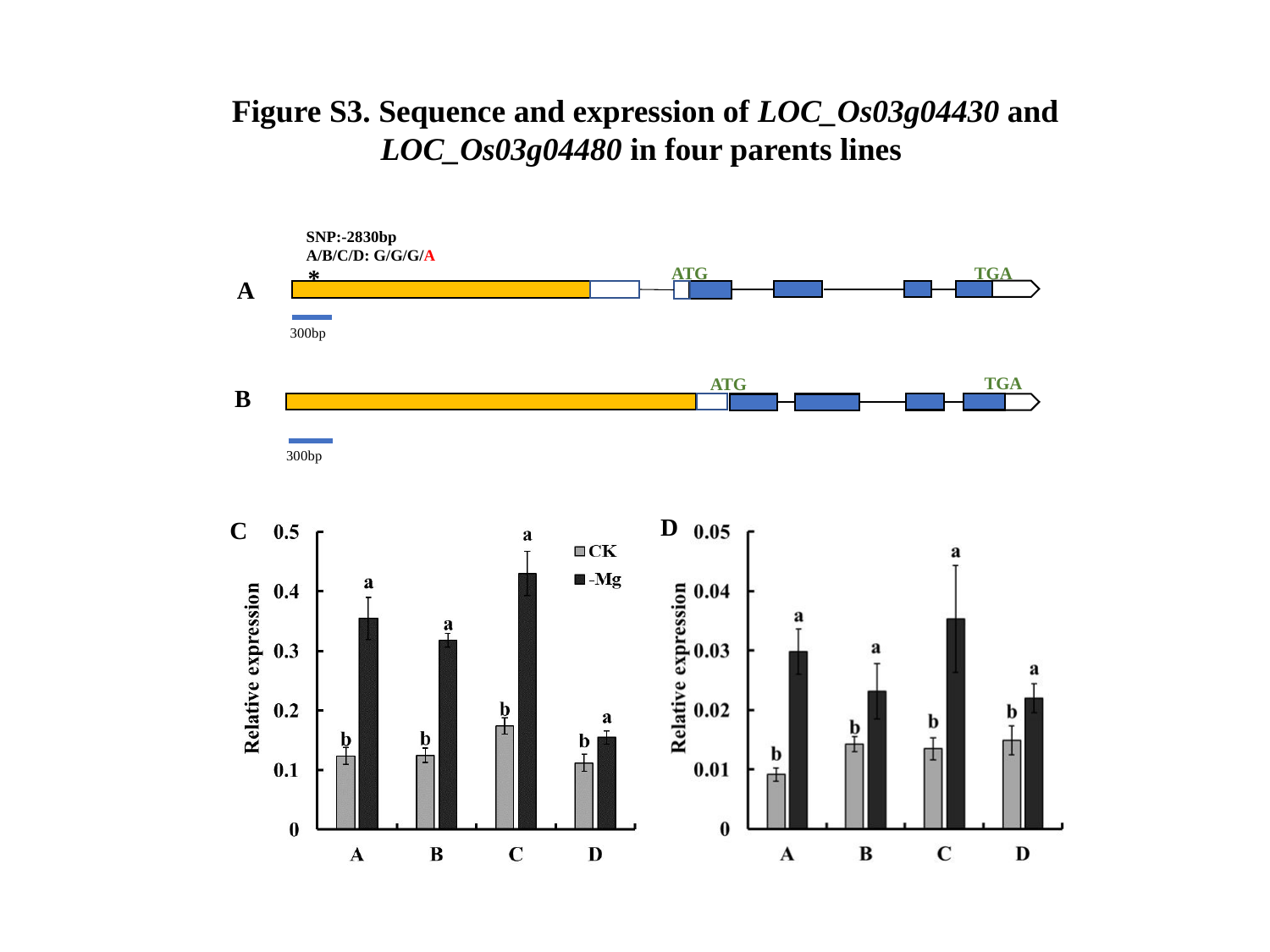

Figure S3. Sequence and expression of LOC_Os03g04430 and LOC_Os03g04480 in four parents lines
SNP:-2830bp
A/B/C/D: G/G/G/A
 ATG TGA
*
A
300bp
TGA
ATG
B
300bp
D
C
